# Every Dog Has Its Data: Evaluation of a Technology-Aided Canine Rabies Vaccination Campaign to Implement a Microplanning Approach

**DOI:** 10.3389/fpubh.2021.757668

**Published:** 2021-11-01

**Authors:** Benjamin Monroe, Fleurinord Ludder, Pierre Dilius, Kelly Crowdis, Frederic Lohr, Julie Cleaton, Luke Gamble, Jesse Blanton, Melissa Etheart, Emily G. Pieracci, Marco Antonio Natal Vigilato, Baldomero Molina-Flores, Max Millien, Andrew D. Gibson, Ryan M. Wallace

**Affiliations:** ^1^Poxvirus and Rabies Branch, Centers for Disease Control and Prevention, Atlanta, GA, United States; ^2^Haiti Ministry of Agriculture, Rural Development and Natural Resources, Port au Prince, Haiti; ^3^Christian Veterinary Mission, Port au Prince, Haiti; ^4^Mission Rabies, Cranborne, United Kingdom; ^5^Centers for Disease Control and Prevention, Haiti Office, Port au Prince, Haiti; ^6^Pan American Center of Foot-and-Mouth Disease and Veterinary Public Health - Pan American Health Organization Veterinary Public Health Unit/(PANAFTOSA/VPH-PAHO), Rio de Janeiro, Brazil; ^7^Division of Genetics and Genomics, Easter Bush Veterinary Centre, The Roslin Institute and the Royal (Dick) School of Veterinary Studies, The University of Edinburgh, Roslin, United Kingdom

**Keywords:** rabies, vaccination, mobile healthcare application, health economic perspectives, mHealth

## Abstract

**Background:** Robust dog vaccination coverage is the primary way to eliminate canine rabies. Haiti conducts annual canine mass vaccination campaigns, but still has the most human deaths in the Latin American and Caribbean region. We conducted an evaluation of dog vaccination methods in Haiti to determine if more intensive, data-driven vaccination methods, using smartphones for data reporting and geo-communication, could increase vaccination coverage to a level capable of disrupting rabies virus transmission.

**Methods:** Two cities were designated into “Traditional” and “Technology-aided” vaccination areas. Traditional areas utilized historical methods of vaccination staff management, whereas Technology-aided areas used smartphone-supported spatial coordination and management of vaccination teams. Smartphones enabled real time two-way geo-communication between campaign managers and vaccinators. Campaign managers provided geographic instruction to vaccinators by assigning mapped daily vaccination boundaries displayed on phone handsets, whilst vaccinators uploaded spatial data of dogs vaccinated for review by the campaign manager to inform assignment of subsequent vaccination zones. The methods were evaluated for vaccination effort, coverage, and cost.

**Results:** A total of 11,420 dogs were vaccinated during the 14-day campaign. The technology-aided approach achieved 80% estimated vaccination coverage as compared to 44% in traditional areas. Daily vaccination rate was higher in Traditional areas (41.7 vaccinations per team-day) compared to in technology-aided areas (26.8) but resulted in significantly lower vaccination coverages. The cost per dog vaccinated increased exponentially with the associated vaccination coverage, with a cost of $1.86 to achieve 25%, $2.51 for 50% coverage, and $3.19 for 70% coverage.

**Conclusions:** Traditional vaccination methods failed to achieve sufficiently high vaccination coverages needed to interrupt sustained rabies virus transmission, whilst the technology-aided approach increased coverage above this critical threshold. Over successive campaigns, this difference is likely to represent the success or failure of the intervention in eliminating the rabies virus. Technology-aided vaccination should be considered in resource limited settings where rabies has not been controlled by Traditional vaccination methods. The use of technology to direct health care workers based on near-real-time spatial data from the field has myriad potential applications in other vaccination and public health initiatives.

## Background

Rabies is one of the most lethal infectious diseases, and is responsible for an estimated 59,000 human deaths worldwide each year (1). Bites from dogs are responsible for ~99% of human rabies deaths, largely in Africa and Asia (2). Dog rabies was successfully eliminated in many Western-hemisphere countries, with the Pan American Health Organization and governments in Latin American countries making drastic reductions in rabies prevalence during recent years through implementation of large-scale and sustained dog vaccination programs (3–5). Investment in dog vaccination is considered the single most effective strategy at reducing rabies disease burden, if the program can achieve adequate coverages to halt enzootic dog-mediated rabies virus transmission (3). Global observations of the relationship between vaccination coverage and rabies incidence in dogs have shown that annual vaccination coverages exceeding 70% of the population are necessary to eliminate or prevent rabies outbreaks (6). However, insufficient resources, incorrect assumptions of dog population sizes, and low community awareness of rabies have chronically challenged the ability to reach these coverages in many endemic countries (7, 8).

The Caribbean nation of Haiti has the highest poverty rate of all countries in the Americas. The resultant socio-economic instability has severely limited the capacity of health and veterinary institutions to respond to public needs (9). Haiti has the highest estimated human rabies death rate in the Western hemisphere and a rate similar to that of most sub-Saharan African countries (1, 10, 11). Previous studies in Haiti indicate that there are frequent exposures to rabid animals, with many people unaware of when and where to receive PEP (11, 12). The majority of dogs in Haiti are reported to be owned-free-roaming or only semi-dependent on human care, therefore allowing for frequent dog-dog interactions and facilitating transmission of infectious agents such as rabies, distemper, and canine transmissible venereal tumor. Ensuring herd immunity among free-roaming dogs is a necessity for ensuring the success of rabies elimination program in settings such as these (13).

Annual dog vaccination campaigns in Haiti have historically immunized an estimated 100,000–300,000 dogs per year. These campaigns have traditionally consisted of vaccination teams operating temporary vaccination clinics in high-profile locations for a short time (i.e., churches, schools, common gathering locations). Only restrained dogs presented by owners are vaccinated before the team moves to another location as attendance wanes. Whilst requiring minimal operational resources to implement, this method likely overlooks a sizable proportion of Haiti's free-roaming dog population.

Traditional vaccination approaches rarely collect operational data to inform subsequent campaigns and make them more effective and efficient. In Haiti, the Traditional vaccination approach relies on vaccinator self-reported daily dog vaccination records. These daily totals are then aggregated for each vaccination locality, then at each department, and finally at the national level. The Traditional approach practiced in Haiti and many other low- and middle-income countries (LMICs) results in poor quality data that can take months to years before it is available for evaluation and to inform future vaccination efforts. This system does not allow for accurate data collection, timely review of data or identification of communities where vaccination rates may have failed to reach intended thresholds. In addition to questionable data quality in many LMIC vaccination campaigns, vaccination coverages are rarely assessed through field-based methods, as recommended by the World Organization for Animal Health (OIE). The combination of poor data and lack of validation of vaccination coverages can lead to chronic under-vaccination and persistent rabies endemicity.

There is a need to develop data-driven strategies for vaccination campaign management in Haiti which maximizes the vaccination coverage and is functional within the limited available resources. A shift toward microplanning for campaign implementation has improved the effectiveness of large-scale immunization initiatives to control polio, cholera, and measles-rubella (14–16). Microplans are developed using campaign data at the village and sub-district levels to identify local challenges and enable campaign managers to implement their own corrective solutions (16). A smartphone application and online management system developed by the global charity Mission Rabies (www.wvsapp.org and http://www.missionrabies.com/app) has been developed to enable efficient, scalable microplan implementation for mass dog vaccination. The system has coordinated the delivery of over one million doses of rabies vaccine to dogs in Africa and Asia (17). The use of the smartphone-based application has the potential to overcome concerns about data quality and vaccination coverage in Haiti, but the feasibility of its introduction was uncertain in this resource-limited country.

This is the first study to formally evaluate whether a smartphone-enabled microplanning methodology can result in improved vaccination coverage at a reasonable level of cost effectiveness. Vaccination coverage estimates from census data and human-dog ratios are also compared to estimates from targeted community dog counts and household surveys immediately after vaccination to compare different interpretations of post-vaccination coverage.

## Methods

### Study Location

**Figure 1:**
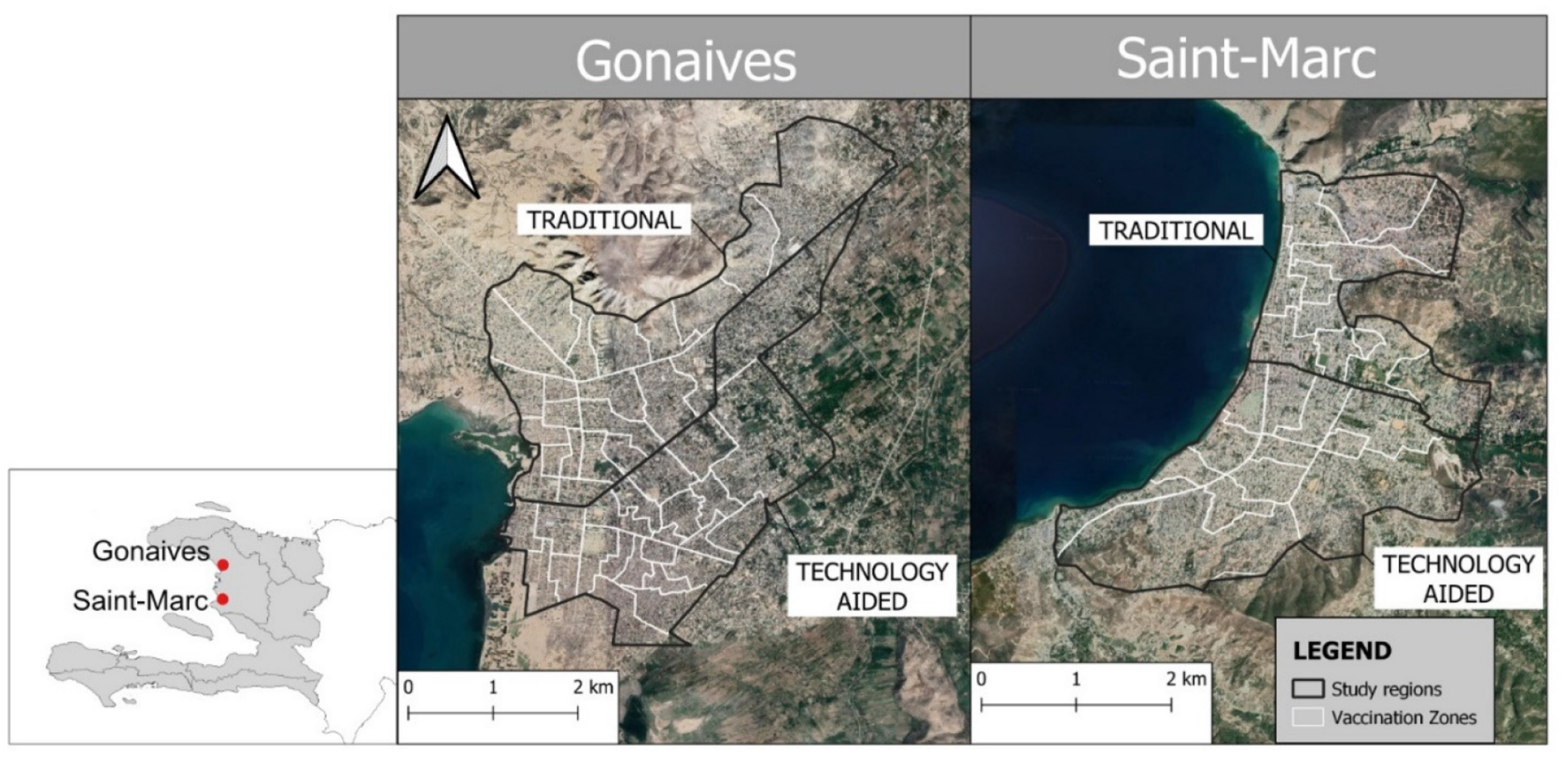
Map showing Gonaives and Saint-Marc study regions (black) and vaccination zones (white). Base map is Google Satellite©. Insert shows the location of the two cities in Haiti.

Two urban (>750 people/km^2^) areas in the Artibonite Department of Haiti: Gonaives and Saint Marc were selected to conduct a case-control evaluation of the use of a smartphone application to implement a microplanning vaccination approach (Figure 1). These locations represent the two largest cities in the Artibonite Department, the second largest department in Haiti (18). These cities were selected out of convenience, as the Haiti Ministry of Agriculture had already planned a vaccination campaign in this department that aligned with the availability of the study coordinators.

### Campaign Planning

The cities of Gonaives and Saint-Marc were divided using major arterial roads to create four distinct study sites of approximately equivalent human and dog populations: Gonaives North, Gonaives South, Saint Marc North, and Saint Marc South. A microplanning approach was applied, whereby these regions were further divided into 69 Vaccination Zones of approximately equivalent area and estimated dog population (Saint-Marc South = 13, Saint-Marc North = 12, Gonaives South = 22, and Gonaives North = 22) (Figure 1). The Vaccination Zones were designed to contain ~400 dogs, the estimated maximum number of dogs that can be completed by two vaccination teams (4 staff, total) working in an area for up to 3 days, based on expert opinion from previous campaigns. Dog population estimates were only used for planning purposes; teams remained in locations until vaccination teams, field managers, or study coordinators decided that adequate vaccination coverage had been achieved. The total number of available vaccination teams for the campaign were divided proportionally between the four study sites so that resources were comparable for the estimated dog population in each study site.

### Data Collection

**Figure 2:**
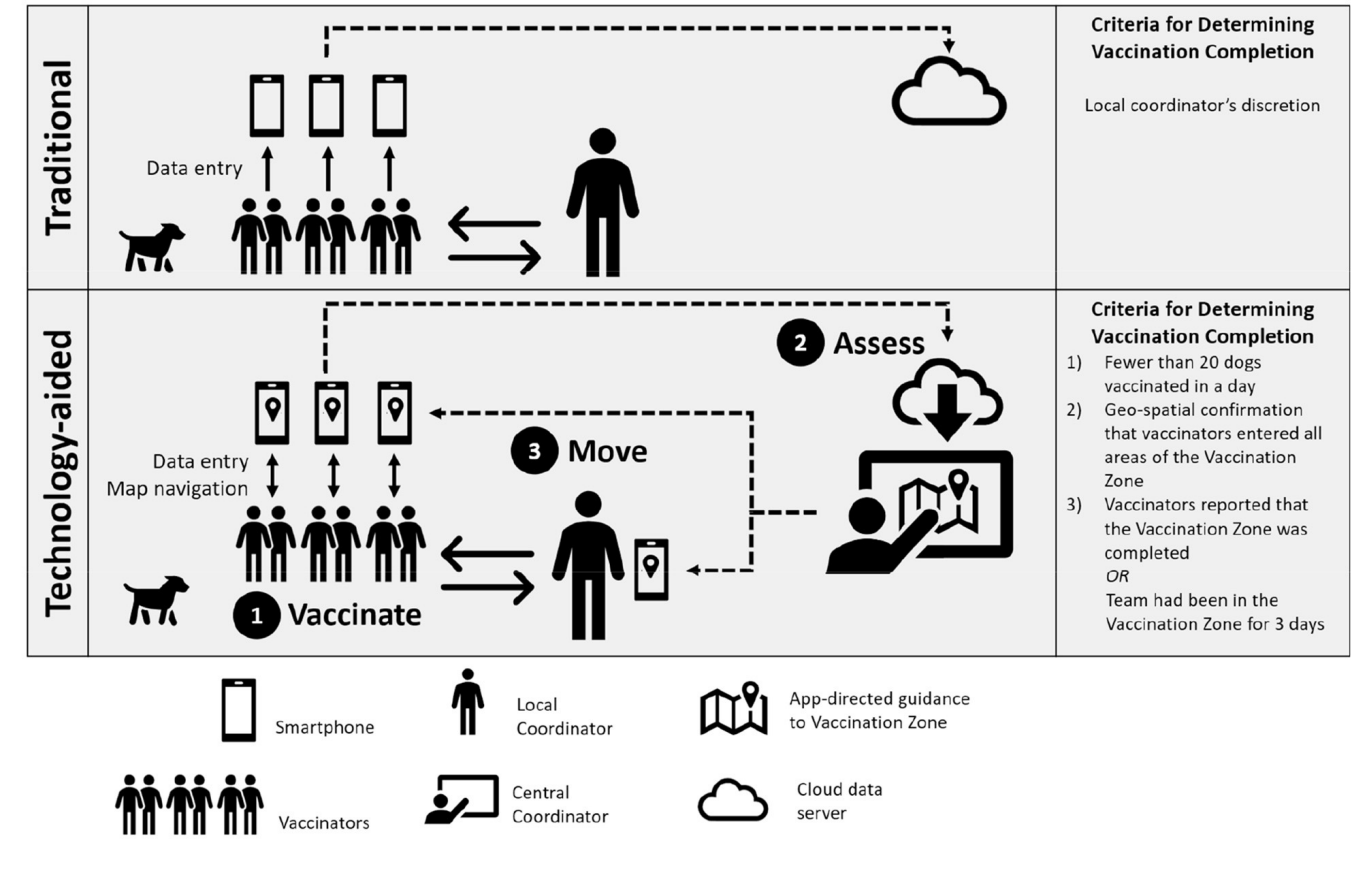
Illustration of the Traditional and Technology-aided methods of campaign management evaluated in the study, including the criteria for guiding team direction by coordinators.

A smartphone-web system was used to collect and aggregate vaccination data from all vaccination teams during the study. The system consists of two components: (1) a field smartphone app (WVS App) and (2) a backend website interface. The WVS app was used by vaccination teams to collect vaccination data, which were then uploaded at the completion of each vaccination day to a secure server where it could be accessed by campaign managers via the website platform for data review and campaign management [Figure 2; (17)]. The dataset included GPS location, time, date, and username for every dog vaccinated. Additional functionalities within the WVS App to support team direction were implemented in certain study areas and formed the basis of evaluation (described below). Data entry into the WVS App was performed offline at the time of vaccination, and submitted via cellular internet connection at the end of the day.

A vaccination team consisted of one veterinary technician and one assistant, with veterinary technicians having at least 2 years of experience operating rabies vaccination campaigns under the direction of the Ministère de l'Agriculture, des Ressources Naturelles et du Développement Rural (MARNDR). Vaccination teams underwent a half-day training on use of the WVS app prior to the campaign. Teams could choose between three vaccination strategies; (1) Static vaccination point (SVP) strategy where community members are encouraged to bring dogs to a fixed location where a temporary vaccination clinic has been established, (2) Door-to-door (DD) strategy where vaccination teams walked through communities inquiring if there were dogs to be vaccinated at each house, (3) Capture-vaccinate-release (CVR) strategy was available on request of the field managers using vaccination teams with additional training and equipment. Teams could switch strategies at their discretion, for example beginning DD vaccinations when attendance at SVP sites began to wane. Vaccinations were performed by trained technicians administering 1.0 mL dose of inactivated cell culture vaccine (Biogenesis Bago, Argentina) subcutaneously. Vaccinated dogs were marked with a temporary wax crayon on the forehead and elastic gauze collar for identification in post-vaccination surveys and prevention of repeated vaccination. Each team received a unique wax color to ensure that dogs were attributed to the correct vaccination method during post-vaccination field surveys.

Vaccination teams were directed by field managers; veterinary agents from the Department of Agriculture Office. These field managers were responsible for the daily management of vaccination teams, including reviewing the area vaccinated each day, managing bite exposures and ensuring vaccination data upload within the WVS App.

### Campaign Management Evaluation

The four study sites were assigned either Traditional (control) or Technology-aided management methodologies (case), resulting in an effective case-control study for the purpose of methodological comparison. Gonaives North and Saint-Marc North were assigned to the Traditional method and Gonaives South and Saint-Marc South were assigned to the technology-aided method; local coordinators considered these cities to have homogenous human populations and identified no concerning differences in population density, economic disparities, or dog ownership characteristics across the regions. Upon completion of the vaccination campaign, post-vaccination surveys were conducted to ensure adequate coverages had been met. In study sites assigned Traditional method, additional vaccination using a Technology-aided method was conducted if post-vaccination evaluation determined that adequate coverage had not been met. Therefore, in total three vaccination methods were evaluated in this study:

1) Traditional vaccination method (Gonaives North and Saint Marc North)
2) Technology-aided vaccination method (Gonaives South and Saint Marc South)
3) Technology-aided after traditional vaccination method (Gonaives North and Saint Marc North)

The campaign was scheduled to run for up to 14 days. Field managers at Traditional vaccination campaigns were able to stop vaccination early if they felt their respective study sites had reached adequate coverages (70%), but were told that there were funds, supplies, and staff available for the entire 14-day period.

#### Traditional Vaccination

Vaccination Zones in Traditional vaccination study sites were vaccinated using campaign management methods that have been used in Haiti for over 10 years, whereby local coordinators selected vaccination sites and directed vaccination teams to community geographic landmarks (i.e., markets, schools, churches, etc.; Figure 2). Each event of a dog vaccination was recorded in the WVS App for analysis purposes although, unlike in technology-aided areas, no feedback or direction was provided through the technology. In effect, data flow was one-way, from the vaccinator to study coordinators. The field manager decided whether to direct each vaccination team to a new location based on paper records of the number of dogs vaccinated and vaccination team feedback.

#### Technology-Aided Vaccination

In technology-aided vaccination study sites, team movements, and monitoring were aided by the WVS App as previously described by Gibson et al. (19). The WVS App enabled two-way geo-communication between central campaign coordinators, local coordinators, and vaccination teams throughout the campaign. Study coordinators accessed the system through a secure web-platform where electronic vaccination records could be reviewed and Vaccination Zones could be assigned to vaccination teams when certain criteria were met. Each vaccination team and field manager carried a smartphone with a user-specific login to the WVS App, in which coordinator-assigned working Vaccination Zones were displayed on Google Maps© and electronic forms enabled entry of vaccination data. After field vaccination data were uploaded at the end of each vaccination day, study coordinators assessed the distribution and intensity of vaccination activity in the backend website interface and re-assigned Vaccination Zones accordingly. At the end of each day, coordinator-assigned Vaccination Zones were pushed out to each vaccination team via the smartphone application. Each morning the field manager and vaccination team would refresh the application to retrieve the daily instructions, thus completing the 2-way communication system. This dynamic, iterative process of vaccinate, assess, move enables the systematic spatial progression of a vaccination team based on vaccination data review in near-real-time (Figure 2).

#### Technology-Aided After Traditional Vaccination (TA-After-Traditional)

In Traditional study sites, after field managers self-determined that the study area was properly vaccinated, a separate team conducted post-vaccination evaluations to determine if adequate coverages had truly been met. If they had not been met, the vaccination teams were re-assigned Vaccination Zones and managed using the technology-aided vaccination method (TA-after-traditional) until the full 14-day vaccination campaign was completed or until post-vaccination surveys found that adequate coverages had been met.

#### Criteria for Technology-Aided Vaccination Zone Completion

Vaccination Zones for the whole campaign area were loaded as a KML file into the web platform before the beginning of the campaign. During the Technology-aided and TA-after-traditional vaccination campaigns, central coordinators assigned Vaccination Zones to vaccination teams following a daily review of electronic records. Teams were assigned a new Vaccination Zone if the following criteria were met (i) fewer than 20 dogs per team were vaccinated in a given day, (ii) geographic visualization of vaccination points indicated homogenous vaccinations within the zone, and (iii) the teams indicated that the Vaccination Zone was complete.

### Public Awareness

All communities were informed of the vaccination campaign from amplified public announcements broadcast by local coordinators while driving through the area several days before vaccination began. Additionally, 600,000 SMS messages were purchased from a major national cellular network provider and distributed to all active customers registered in the two cities at the onset (initial 300,000 messages) and midpoint (final 300,000 messages) of the campaign. The message, in the Haitian Creole language, encouraged residents to bring all dogs to the free vaccination campaign. An evaluation of the SMS-based communication campaign has been published elsewhere (20). Vaccination teams were also equipped with megaphones to announce their presence in a neighborhood and encourage participation. Vaccination was free to dog owners. There were no differences in awareness effort conducted across the four study sites.

### Vaccination Coverage Assessment

Vaccination coverage was estimated in each study site using three methods; dog sight surveys, household surveys, and human:dog ratio (HDR).

#### Field Based Vaccination Coverage

Dog sight surveys were conducted in a sample of Vaccination Zones to estimate vaccination coverage in the free-roaming dog population. A two-stage cluster-sampling design was used to randomly select 33% of Vaccination Zones for post-vaccination surveys. Dog sight surveys were completed over 2 consecutive days to allow for a sight/re-sight analysis using the Lincoln-Petersen formula, which has been described in detail in other publications (21, 22). Free-roaming dog vaccination coverage was determined by calculating the proportion of dogs marked as vaccinated from all dogs seen, and adjusted for collar-loss to ensure comparability across study sites (22, 23). Field survey teams were independent from the vaccination teams to ensure that no bias was introduced through knowledge of the areas that had been vaccinated.

Dog sight surveys were not conducted after the TA-after-traditional vaccinations. To approximate this coverage, we applied the following equation:


$$\begin{array}{l} {}[(N(TAaT\;dogs\;vaccinated)+N(TA\;dogs\;vaccinated))/\\ \;N(TA\;dogs\;vaccinated){]}^{*}\;TA(Field-Based) \end{array}$$

Where *N* = *Number, TAaT* = *Technology-aided after Traditional*, and *TA* = *Technology-Aided*

#### Household Surveys

Household surveys were conducted in regions selected for dog sight surveys to estimate vaccination coverage in the owned dog population. Vaccination coverage and human-dog-ratios (HDR) were determined from household interviews using a standardized questionnaire administered to consenting adults (>18 years). Post-vaccination household surveys were initiated in a Vaccination Zone once the field managers indicated they were complete. Owned-dog vaccination coverage was the total number of dogs reported as vaccinated during the campaign by the owner, out of the total dog population reported as owned by survey respondents. Proof of vaccination was not required.

The sample size for the household interviews was calculated from an estimated human population of 291,500 in the full campaign area. It was estimated that 50% of households in Haiti own dogs (13). The household survey sample size of 634 was derived from an alpha = 0.05, a design effect of 1.5, and a 10% non-response rate. Survey sample size was distributed across the four study sites, proportional to the human population, and a skip pattern was derived based on the number of households in the site. Interviews were conducted in Haitian Creole by trained interview teams reading aloud from a script and recording answers on an electronic form in the WVS App and as for dog sight surveys, staff were independent of vaccination teams. Informed consent was obtained from each person prior to beginning the interview.

#### Population-Based Vaccination Coverage

The mean city-wise HDR estimate from household surveys was used to estimate the total dog population in each study site. Dog vaccination coverage was estimated using the total number of vaccinations by study site as the numerator and the HDR estimated dog population as the denominator.

#### Data Analysis

All manipulation and analysis of geographic information was performed using ArcMap 10.5 (ESRI, Redlands CA, USA). At the completion of the campaign, a final dataset was aggregated from all data uploaded to the web platform and checked for accuracy against paper records. Daily and hourly vaccination rates for teams were calculated from the timestamps associated from each vaccination record using Microsoft Excel 2016 (Microsoft Inc., Redmond WA). Tests of significance for comparisons of daily mean vaccinations by area and method were calculated with an ANOVA in EpiInfo 7 (Centers for Disease Control and Prevention, Atlanta, USA). A CSV file containing the geographic coordinates of all vaccination points were spatially joined to a layer containing vaccination zone boundaries in ArcMap 10.5 to determine population-based vaccination coverage estimates.

Costs were calculated for the following categories: education/awareness, transportation/logistics, vaccines/consumables, and vaccinator salary. Since vaccination study sites were not of equal sizes, the overall costs attributed to each site were adjusted based on the study site human populations and calculated as follows:

Daily Campaign Cost: C(Daily Awareness)^*^(Site Population / Full Study Population) + C(Daily Logistical Costs)^*^(Site Teams / Full Study Teams) + C(Daily Salary)^*^N(Site Teams) + N(Vaccinated Dogs)^*^C(Vaccines)


$$\begin{array}{l} Where:C=cost\;and\;N=Number \end{array}$$

Costs per dog vaccinated were calculated for each study site. The cost per 1% increase in the field-based dog vaccination coverage achieved was calculated by dividing the total in-category vaccination costs by the total vaccination coverage. This is meant to approximate the program expenditures to reach a desired vaccination coverage level. Since the coverage was not assessed for TA-after-traditional vaccination, the field-based vaccination coverage was adjusted proportionally with the number of additional dogs vaccinated by this method. The average cost per dog vaccinated to achieve 70% coverage was calculated and compared across methods.

### Results

#### Canine Mass Vaccination

Vaccination Zones had a mean area of 0.33 km^2^ (range 0.06–1.65 km^2^) and an average dog population estimate of 408 (range 257–574) based on reported human population and human-to-dog ratio of 14:1 derived from study results. On average, 29 teams (58 vaccination staff) were active each day and the entire vaccination campaign involved 422 vaccination team-days from May 20 to June 5, 2017.

**Figure 3:**
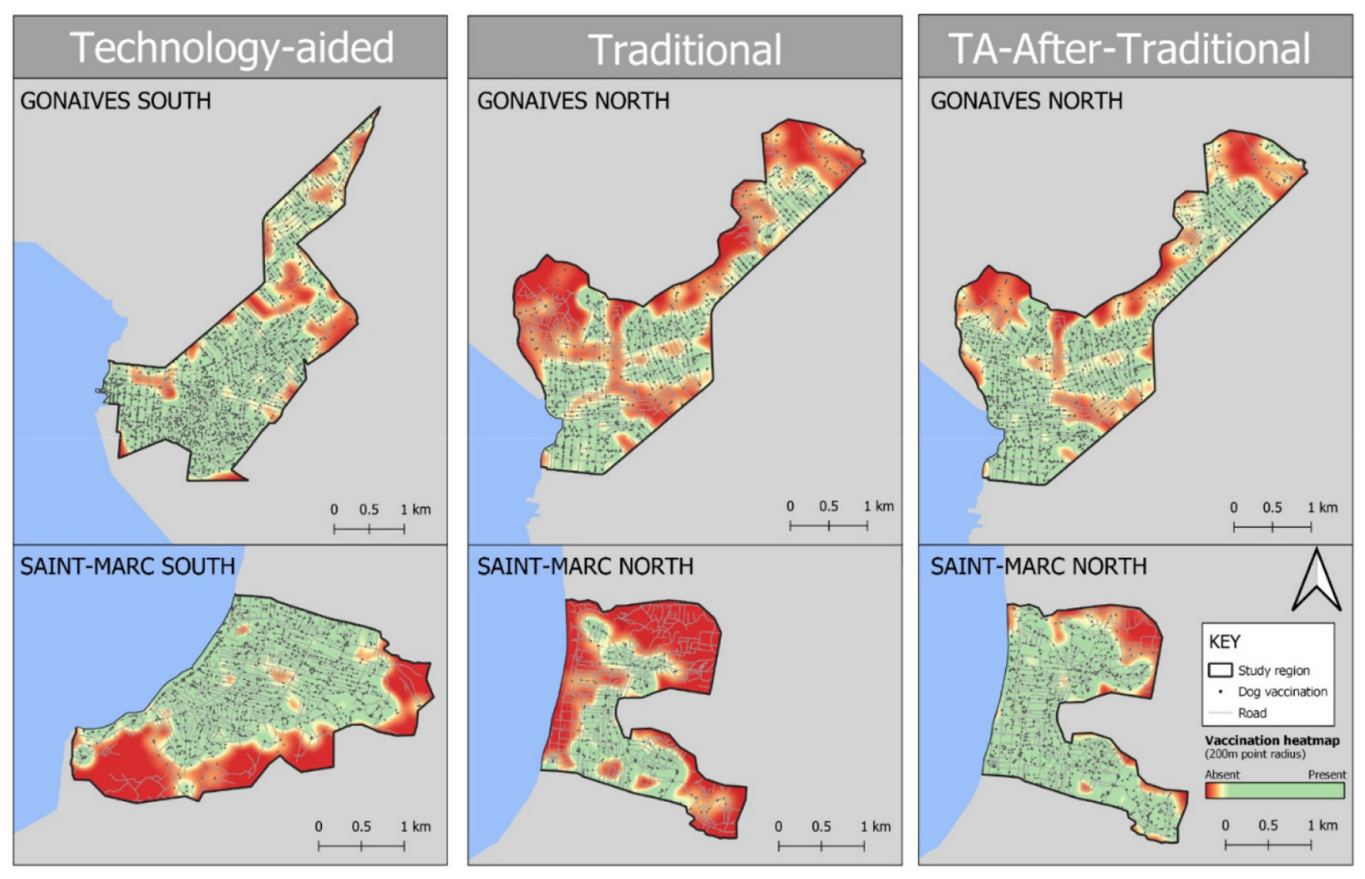
Heatmap of vaccination distribution in study regions by vaccination methodology in Gonaives and Saint-Marc, Haiti. Individual vaccinations are indicated by gray points and roads by gray lines (Open Street Map). Heatmap is set to a radius of 200 m to indicate presence or absence of vaccination effort across the region.

A total of 11,420 dogs were vaccinated during the campaign, an overall average of 816 vaccinations per day (range = 358–1,297) (Figure 3). On 19 occasions (4.5% of daily uploads), vaccination records for the day could not be uploaded to the server due to internet connection errors; for the 19 team-days with no vaccination data, the overall daily average across all sites was imputed and applied. Information on the vaccination strategy used was available for 92% of daily vaccination records (*n* = 10,503). The door-to-door (DD) vaccination strategy accounted for 80.7% (*n* = 8,475) of all vaccinations, with static vaccination point (SVP) and capture-vaccinate-release (CVR) accounting for 19.6% (*n* = 1,868) and 1.2% (*n* = 160), respectively.

##### Traditional Vaccination Method

Field managers declared areas assigned the Traditional vaccination method of Gonaives and Saint Marc complete after seven and three campaign days (67 and 15 team-days), respectively. The mean rate of vaccination by the Traditional method was 41.7 dogs per team-day.

##### Technology-Aided Vaccination Method

Teams in the technology-aided regions vaccinated for all 14 days of the campaign, representing 135 and 84 vaccination team-days in Gonaives and Saint Marc, respectively. Teams conducting the technology-aided vaccination method vaccinated 26.8 dogs per team-day (Table 1).

**Table 1:** Summary characteristics for each vaccination area.

**Vaccination method^a^**	**Traditional (North)**			**Technology-aided (South)**		
**Location**	**Gonaives**	**Saint Marc**	**Total**	**Gonaives**	**Saint Marc**	**Total**
Estimated human population	86,000	42,500	128,500	101,000	62,000	163,000
Pre-study estimated dogs (10:1 HDR)	8,565	4,442	13,007	10,028	5,135	15,163
Total Vaccination Zones	22	12	34	22	13	35
Evaluated Vaccination Zones	7 (31.8%)	3 (25.0%)	10 (29.4%)	8 (36.4%)	4 (30.8%)	12 (34.3%)
Mean Vaccination Zone Area (km^2^)	0.31	0.30	0.31	0.31	0.44	0.38
Mean number of dogs/Vaccination Zone	390	370	380	456	395	426
Vaccination teams	9	5	14	9	6	15
Estimated dog population per vaccination team	952	888	920	1,114	856	924
**Campaign days used^b^**	**Traditional (North)**			**Technology-aided (South)**		
Traditional	7	3	10	0	0	0
Technology-aided	7	11	18	14	14	28
Total	14	14	28	14	14	28
**Vaccination team-days used^c^**
Traditional method	67	15	82	0	0	0
Technology-aided method	66	55	121	135	84	219
Total	133	70	203	135	84	219
**Total vaccinations**
Traditional	2,717	702	3,282	0	0	0
Technology-aided	965	1,163	2,128	3,766	2,107	5,873
Overall	3,682	1,865	5,410	3,766	2,107	5,873
**Daily mean vaccinations per team**
Traditional	40.6	46.8	41.7	0	0	0
Technology-aided	14.6	21.1	17.6	27.9	25.1	26.8
Overall	27.7	26.6	27.3	27.9	25.1	26.8

a *In a Traditional vaccination method site, local coordinators subjectively decided where to place teams in the area with advanced notice and determined when vaccinations were complete. For the Technology-aided method teams stayed in a defined zone for 2–3 days until no additional dogs were available or vaccinations dropped below 20 per day*.

b *Number of days vaccination teams were active before they were considered complete by local coordinators. Teams in the Technology-aided method areas were active for all 14 campaign days*.

c *Team-days are the sum of all vaccination teams used each day*.

##### Technology-Aided After Traditional Vaccination Method

After local coordinators declared Traditional study sites complete, the Technology-aided method was implemented for the remaining campaign days to increase vaccination coverage in deficient zones (7 days in Gonaives and 11 days in Saint Marc) (Table 1). The rate of vaccination during the Technology-aided method conducted after traditional vaccination was completed was only 17.6 dogs per team-day.

### Vaccination Coverage Assessment

#### Household Survey Vaccination Coverage Estimate

Household survey teams visited 992 households and completed surveys from 682 eligible households (participation rate 68.8%) with 219 households (32.1%) reporting dog ownership. Households reported owning 325 dogs and had a human household population of 4,682, resulting in a study-derived HDR of 14:1 (95% CI 12.6–15.7). The most common confinement status was free roaming (64.6%, *n* = 210), followed by partially confined (27.1%, *n* = 88) with only 8.3% (*n* = 27) of dogs reported to be always confined at home or on a leash.

Surveys found that 66.1% (95% CI 52.9–81.7%) of the 87 dog-owning households surveyed in the Traditional method area reported vaccinating their dogs in the current campaign (Table 2C). In Technology-aided study sites, household surveys found that 72.6% (95% CI 61.6–85.2%) of the 132 dog-owning households surveyed reported their dogs had been vaccinated during the recent campaign (Table 2C).

**Table 2:** Dog counts and vaccination coverage estimates by vaccination area and methodology.

**Location**	**Traditional (North)**			**Technology-aided after traditional**			**Technology-aided (South)**		
	**Gonaives**	**Saint Marc**	**Combined**	**Gonaives**	**Saint Marc**	**Combined**	**Gonaives**	**Saint Marc**	**Combined**
**(A) POPULATION BASED COVERAGE (HDR)**									
Dog estimate (14:1)	6,143 (5,478–6,826)	3,036 (2,707–3,373)	9,179 (8,185–10,199)	6,143 (5,478–6,826)	3,036 (2,707–3,373)	9,179 (8,185–10,199)	7,214 (6,433–8,015)	4,429 (3,949–4,921)	11,643 (10,382–12,937)
Vaccinated dogs	2,717	702	3,419	3,682	1,865	5,547	3,766	2,107	5,873
Coverage	44.2% (39.8–49.6)	23.1% (20.8–25.9)	37.2% (33.5–41.8)	59.9% (53.9–67.2)	61.4% (55.3–68.9)	60.4% (54.4–67.8)	52.2% (47.0–58.5)	47.6% (42.8–53.4)	50.4% (45.4–56.6)
**(B) FIELD BASED COVERAGE**									
Total dogs sighted	187	196	383	–	–	–	414	136	550
Marked dogs	118	50	168	–	–	–	339	103	441
Coverage^*^	63.1% (56.0–69.7)	25.5% (19.9–32.0)	43.9% (39.0–48.9)	86%^**^	68%^**^	72.4%^**^	81.9% (77.9–85.3)	75.7% (67.9–82.2)	80.2% (76.7–83.3)
**(C) HOUSEHOLD SURVEY COVERAGE**									
Reported owned	33	91	124	–	–	–	125	76	201
Reported vaccinated	17	65	82	–	–	–	86	60	146
Coverage^*^	51.5% (31.0–80.8)	71.4% (55.6–90.5)	66.1% (52.9–81.7)	–	–	–	68.8% (55.4–84.6)	79.0% (60.8–100.0)	72.6% (61.6–85.2)

* *Confidence intervals for field-based and household survey vaccination coverages were calculated using the Wilson Score corrected for population size*.

** *Coverage estimates were calculated based on the increase in number of dogs vaccinated during the Technology-aided-after-traditional method was conducted. Post-vaccination surveys were not conducted after this method was performed*.

#### Population-Based Vaccination Coverage Estimate

Using the HDR of 14:1 from the household surveys, dog population estimates yielded a mean estimated vaccination coverage of 37.2% following the Traditional vaccination method (Gonaives North = 44.2%, Saint Marc North = 23.1%) (Table 2A). After teams completed the TA-after-traditional vaccination method in these regions, this rose to 60.4% (Gonaives North = 59.9%, Saint Marc North = 61.4%). In Technology-aided study sites the coverage estimate using the HDR post-vaccination evaluation method was 50.4% (Gonaives South = 52.2%, Saint Marc South = 47.6%).

#### Field Based Vaccination Coverage Estimate

In Traditional study sites 2-day post-vaccination field surveys sighted 383 free-roaming dogs in the 11 selected Vaccination Zones. Of these dogs, 43.9% (95% CI 39.0–48.9%) had evidence of vaccination (Table 2B). After teams completed the TA-after-traditional vaccination method in these regions, this rose to 72.4% (Gonaives North = 86%, Saint Marc North = 68%). In Technology-aided study sites, post-vaccination dog sight surveys sighted 550 free-roaming dogs in 13 selected Vaccination Zones, of which 80.2% (95% CI 76.7–83.3%) had evidence of vaccination. Field-based evaluations were not conducted after the TA-after-traditional method.

When comparing between respective study sites and post-vaccination evaluation methods, the Technology-aided method resulted in higher vaccination coverages as compared to the Traditional method. In Gonaives, the Technology-aided method resulted in 1.2-fold greater overall vaccination coverage as measured by the population-based and 1.3-fold greater free-roaming dog vaccination coverage as measured by field surveys. In Saint Marc, the Technology-aided method resulted in 2.1-fold greater overall vaccination coverage as measured by the population-based and 1.8-fold greater free-roaming dog vaccination coverage as measured by field surveys. There was no difference in reported owned-dog vaccination coverage as measured by the household survey method in Saint Marc or Gonaives (*P* = 0.89). Overall, the Technology-aided study sites had significantly higher population-based and free-roaming dog vaccination coverages as compared to the Traditional vaccination sites (*p* < 0.001). After implementing TA-after-traditional, an additional 2,128 dogs were vaccinated; a 62% increase in the number of dogs vaccinated resulting in a significantly higher dog vaccination coverage as measured by the HDR evaluation methods (60.4 vs. 37.2%, *p* < 0.001).

## Cost of Vaccination

The total cost estimate for all campaign areas and methods was $24,932 ($2.18 per dog vaccinated). Approximately $10,721 (43%) of the dog vaccination budget was spent on awareness (primarily text messages) ($112–$265 per site operational day). The remaining $14,211 was spent on consumables ($0.30 per dog), transportation ($50–$89 per team-day), and labor ($16.00 per team-day). In addition to these routine costs, the cost for data over the 2-week campaign period was $2 USD per team, or $0.14 per team-day). This cost is less than one penny per dog recorded in the App ($0.005 USD). Furthermore, the mobile phones ($80 per phone) were used for the duration of the national campaign, which recorded 330,000 dog vaccination, for a cell-phone related cost of $0.024 per dog vaccinated. The technology-related costs for this campaign totaled $0.03 USD per dog vaccinated.

**Figure 4:**
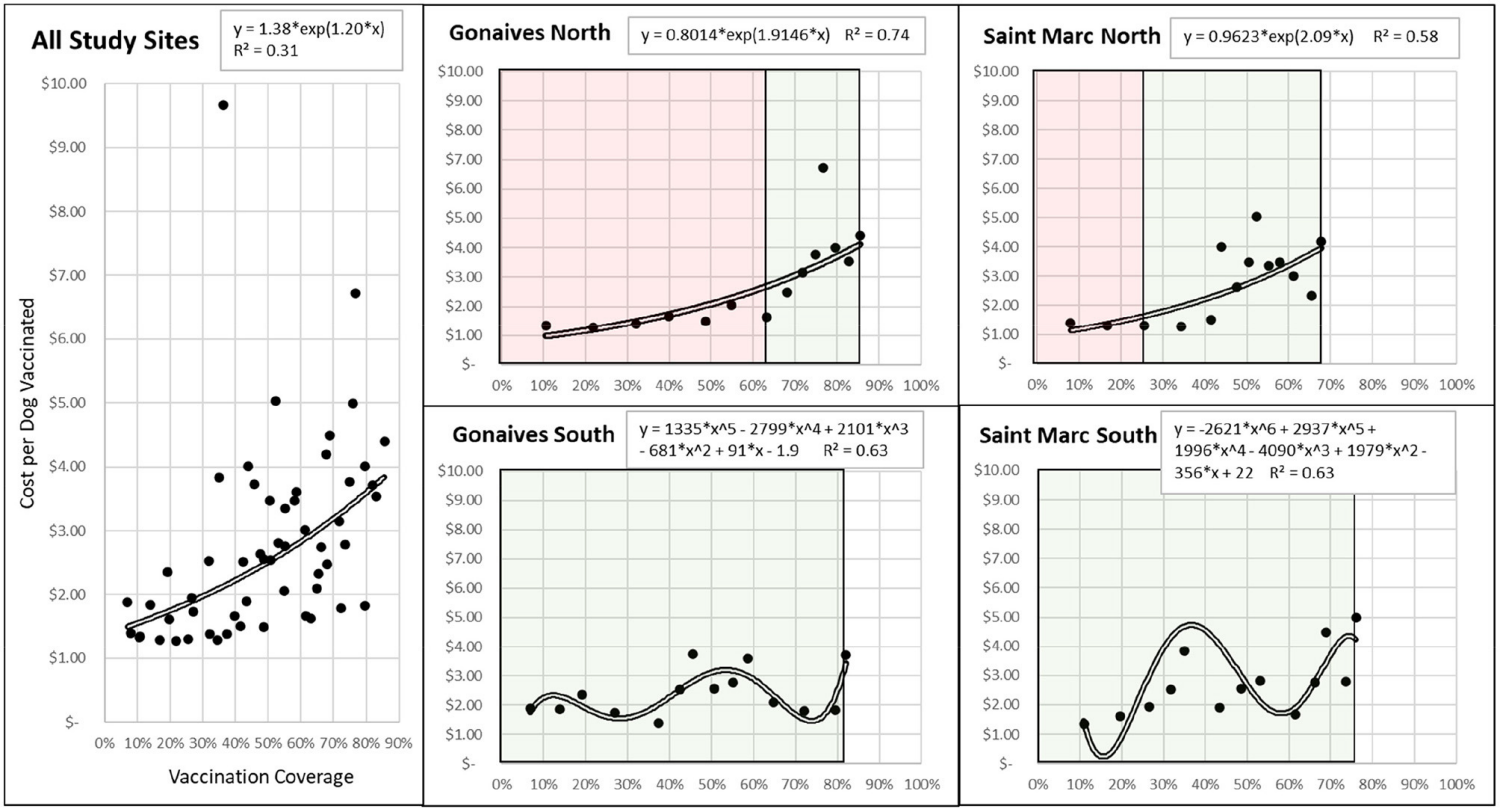
Associations between vaccination coverage and cost per dog vaccinated during a 14-day dog mass vaccination campaign, by site and method. Black dots reflect a daily average cost per dog vaccinated at the respective vaccination coverage. Double black lines represent trendlines reflective of the functional association that explained the greatest degree of variance in the vaccination data (*R*^2^). Equations and *R*^2^-values are provided for comparison. Red background color represents vaccinations conducted by Traditional methods, while Green background color represents vaccinations conducted by the Technology-aided method.

Among the Traditional method sites, Gonaives North averaged $1.66 per dog vaccinated by the Traditional method to achieve a coverage of 63%; this increased to $3.68 per dog vaccination after switching to TA-after-traditional, to increase the vaccination coverage to 86% (Figure 4). At this site, it cost an average of $88 for each 1% increase in dog vaccination coverage. Saint Marc North averaged $1.33 per dog vaccinated by the Traditional method to achieve a coverage of 25.5%; this increased to $2.58 per dog vaccinated by the TA-after-traditional method, which led to a total study-site vaccination coverage of 68%. At this site, it cost an average of $58 for each 1% increase in dog vaccination coverage.

Among Technology-aided sites, Gonaives South averaged $2.19 per dog vaccinated to achieve a coverage of 82%. This site cost an average of $99 for each 1% increase in dog vaccination coverage. Saint Marc South averaged $2.42 per dog vaccinated to achieve a coverage of 76%. This site cost an average of $67 for each 1% increase in dog vaccination coverage.

Overall, the association between the cost per dog vaccinated and the vaccination coverage had an exponential association, with higher coverages coming at greater relative costs (Figure 4). The Traditional vaccination method sites showed a strong exponential association where costs increased to achieve higher vaccination coverages, with *R*^2^-values of 0.74 and 0.58 for Gonaives and Saint Marc, respectively. Technology-aided sites showed a polynomial association, likely reflective of the microplanning approach that was enacted. The *R*^2^ of the polynomial functions was 0.63 and 0.34 for Gonaives and Saint Marc, respectively.

## Discussion

This study demonstrates that using technology-aided vaccination is feasible in a resource-limited country and can lead to significant improvement in vaccination coverage while reducing the cost per dog vaccinated to consistently achieve at least 70% vaccination coverage.

Annual dog vaccination campaigns to prevent rabies have been routinely conducted in Haiti for decades, but the persistence of human rabies cases justifies the continued evaluation and improvement in the dog vaccination methods used, and their resulting coverage in the susceptible dog population. This study demonstrates that vaccination coverages achieved by Traditional vaccination methods fell far short of that needed to control rabies, a similar finding to other studies conducted in Haiti (24, 25). However, the target of 70% vaccination coverage among free-roaming dogs was exceeded with the Technology-aided approach, using smartphone technology to implement vaccination micro-plans at the sub-village level.

The Technology-aided method evaluated in this study leveraged the improved communication afforded by smartphone-technology to direct vaccination teams on the ground based on incoming dog vaccination data. The result was a more thorough, intentional deployment of resources based on the distribution of the dog population, ultimately achieving considerably higher vaccination coverages. The daily cost per dog vaccinated using the Technology-aided method was relatively constant and fluctuated approximately every 3 days in-line with team movements (Figure 4). While it was not measured in this study, the multi-day strategy enforced by the Technology-aided strategy likely resulted in increased reliance on DD vaccination methods; in addition to remote direction for vaccination locations, the DD strategy also may have improved the vaccination coverage. Although the Traditional method returned a higher daily vaccination rate and was lower in cost per dog vaccinated, the low vaccination coverage reported in this and other studies would be unlikely to realize the goal of canine rabies elimination [Table 3; (13, 26)]. This outcome is reflected in the persistent canine and human rabies cases in Haiti despite decades of dog vaccination campaigns (27) and negates the cost-efficiencies of such an approach.

**Table 3:** Mean daily vaccination counts per team for Traditional, Technology-aided, and Technology-aided after traditional vaccination methods.

	**TA-after-traditional^b^**	**Technology-aided^a^**
Day 1	17.7	33.7
Day 2	14.5	25.7
Day 3	11.3	18.1

a *In areas using the Technology-aided method teams stayed in a defined area and vaccinated for up to 3 days until a target number of dogs was reached*.

b *Technology-aided after traditional (TA-after-traditional) involved initiating the Technology-aided method after a Traditional area was declared complete by a coordinator. With the Traditional method coordinators can choose where teams are placed and for how long*.

Compensating for areas of low coverage in Traditional vaccination study sites through follow-up Technology-aided methods was more expensive per dog vaccinated than Technology-aided methods alone. Furthermore, returning to re-vaccinate in these areas with more intensive methods can be operationally complex. Reduced efficiencies result from difficulties in identifying previously vaccinated dogs when returning to a community a second time, waning community sensitization over time, and logistical challenges to identify and redeploy vaccination teams in the community. In this study, reaching the recommended coverage level of 70% among free roaming dogs was only achieved when the Technology-aided method was implemented and suggests that planning and implementing a Technology-aided approach from the start would be more efficient than first conducting Traditional methods, followed by Technology-aided approaches to mop-up areas of low coverage.

Besides overall mean vaccination coverage, the spatial distribution of vaccine across a population has been shown to be significant to the probability of achieving rabies virus elimination. Heterogeneous vaccination coverage creates an environment where rabies virus transmission can continue in pockets of low vaccination coverage, thwarting the success of the campaign in achieving rabies virus elimination (27, 28). The Technology-aided method not only enabled a higher mean vaccination coverage within the target dog population, but also aimed to avoid patchy vaccination coverage. This was facilitated through the assignment of vaccination teams to defined Vaccination Zones in which they worked over several days, uploading spatial data for each dog vaccinated. Zone re-assignment was based on the number of dogs vaccinated, the geospatial distribution of vaccinations within the zone, and the subjective feedback of the vaccination team; data for which were collected daily in the App. The method prompted teams to return to specific areas where dogs were initially unavailable or to reach pockets of the Vaccination Zone that were missed during earlier visits, therefore increasing the likelihood of homogenous vaccination coverage throughout the region (Figure 3). With the Traditional approach, vaccination teams moved through the community more quickly and selected vaccination locations based on high-profile locations such as churches, schools, and stadiums and the field managers' subjective recollection of past campaigns. The resulting heterogeneous vaccine distribution within the dog population is likely to further hamper the chances of achieving rabies elimination (27, 28).

Traditional campaigns used fewer than half of the total allotted vaccination-days, despite coordinator awareness that there were supplies, staff, and funding available for the full campaign period, an indication that coordinators thought Traditional methods yielded an acceptable coverage level. This highlights the importance of periodic post-vaccination surveys to confirm vaccine program operator decisions on when coverages have been met, as is recommended by OIE. The vaccination program in Gonaives North (Traditional) achieved good coverage by the Traditional method, alone. However, this campaign still failed to achieve the goal of 70% coverage at the time in which the coordinator ended the campaign. This indicates that in certain settings, Traditional vaccination approaches may perform quite well and at a relatively low cost. In the absence of mobile technology to track vaccinations, post-vaccination surveys are required to elucidate the vaccination coverages achieved. This study shows the heterogeneity and unreliability in Traditional vaccination approaches in Haiti, which can be overcome by the implementation of mobile technologies.

Robust evaluation of dog vaccination coverage is a challenge in resource-limited settings like Haiti. To start, even establishing a denominator for determining accurate coverage estimates in areas where dog population data is incorrect or missing is a difficult task (7). This study compared three methods of calculating vaccination coverage to determine if an appropriate level of herd immunity had been achieved. Human population-based coverage levels, which use human census data and human-to-dog ratios, provided the lowest, and least reasonable, estimates of coverage in the study area. Despite past studies suggesting that urban communities like these might have an HDR of 10:1, results from the household survey conducted in these specific communities indicate that the HDR was higher, at 14.0. This difference in HDR can significantly change the resulting dog vaccination coverage calculation when using a population-based approach. Furthermore, this population-based evaluation method is prone to error when the underlying human census population is inaccurate or outdated. In these communities there is concern that the 2015 national census may portray an inflated human population in these selected communities, as there is thought to be a general population migration to the capital city from these outlying urban communities. The HDR dog vaccination evaluation method is likely the least accurate for determining the total vaccination coverage, but it does allow for direct comparisons across all vaccination methods. In this sense, the HDR post-vaccination evaluation method confirmed that the Technology-aided and TA-after-traditional methods both provide significantly greater vaccination coverage as compared to Traditional method.

Vaccination coverages calculated from field dog-sight surveys, which reflect coverage in the free roaming dog population and are a direct measure of the vaccination efforts, were consistently higher when using the Technology-aided vaccination method and exceeded the target vaccination coverage (70%) in both cities where it was conducted. The field survey method for estimating vaccination coverage is a direct observation method and should result in a more accurate assessment of the coverage in the dog population that is highest priority to vaccinate. The household survey method, which represents the owned dog population, was similar between the Technology-aided and Traditional vaccination methods. This could indicate that owned dogs are just as likely to be vaccinated by each method. It could also suggest that dog owners inaccurately report their participation in dog vaccination campaigns either due to confusion, concern about legal implications, or embarrassment for not vaccinating their dogs. Several studies have conducted household post-vaccination surveys in which owners are required to show proof of vaccination to verify the coverage estimates, which can eliminate this potential bias (29, 30). Haiti does not routinely provide proof of vaccination, so this was not possible under the current study design.

Final vaccination coverage by dog enumeration in technology-aided vaccination areas was almost twice what was seen in the Traditional areas, 80.2 and 43.9%, respectively. This difference may be due to the principle that the Traditional vaccination strategy mainly targets owned dogs which are confined at the time vaccinators present in the community and are readily able to be presented to the vaccination teams. Traditional methods bias vaccine access to well-owned, primarily confined dogs. As a result, this method may reach relatively high numbers of dogs, while still failing to achieve herd immunity in the dog populations that are primarily responsible for dog-to-dog enzootic rabies virus transmission. The technology-aided strategy involves returning to the same communities over multiple days, which we believe would allow more opportunities for vaccinating owned, free-roaming dogs that might not have been available for initial vaccination but later confined by owners until vaccination teams returned. The goal of mass dog vaccination campaigns is to establish a high level of herd immunity among the susceptible dog population, that is, dogs that can interact with each other. In this sense, vaccination coverages among the free-roaming dog population are likely the best indicator of a successful campaign, and support that the Technology-aided method described here is more effective at controlling dog-mediated rabies in Haiti.

Routine recording of the GPS location of every vaccination enabled a degree of transparency and accountability impossible to achieve through paper-based records. In addition to recording data, the app allowed the teams to communicate and provide feedback to coordinators regarding the successes and challenges they faced each day. For example, teams could indicate to coordinators when they thought the Vaccination Zone was completed, or could alert coordinators of notable events, such as a dog bite incident. While this data is often communicated during Traditional methods, systematic collection enabled coordinators to access this data more readily in daily plans and to distribute this workload across multiple coordinators who have computer and internet access. Micro-planning vaccination campaigns requires that teams were able to both visualize their Vaccination Zones as well as provide this daily communication to coordinators; a process that was much more feasible with the use of a smartphone app.

Integrating smartphone data collection into a dog vaccination campaign created several logistical challenges. Purchasing the phones increased the costs associated with running the campaign and required additional training time for the vaccination teams to become proficient with the software. However, the same phones were reused for the remainder of the national campaign, which logged over 300,000 dog vaccination records over the following 12 months and are intended to be used for multiple years thereby reducing this cost long term. The discounted cost of phones for large-scale dog vaccination campaigns makes the cost practically negligible compared to the logistical complexities of managing paper-based records. Vaccinators reported that it was sometimes difficult to read the screen in bright daylight and the phones had to be recharged every night for use the next day, creating additional logistical considerations for the campaign coordinators in communities where electricity is inconsistent. Many vaccinators had not navigated using web-based maps and initially found it difficult to find and stay within the boundaries of their assigned Vaccination Zone. When considering the cost to operate the Technology-aided program, the cost of Study Coordinator's time to review daily vaccination records was not considered, nor are the costs to develop or maintain the App. Mission Rabies offers the App free-to-use to collaborators, so it would be unlikely that these costs would be passed on to a low-income country's dog vaccination program. Technical experts time to review and advise on the campaign can be done remotely and could leverage international experts that volunteer their time to assist in vaccination campaigns. The cost for this component is dependent on the design of the campaign and contributions from collaborators, but in certain situations there could be added costs to the program that are not reflected in this analysis. Despite these problems experienced when introducing a new technology-driven approach to dog vaccination, the campaign experienced few problems and a low rate of vaccine data upload failure.

## Conclusions

At the conclusion of this evaluation, national coordinators from MARNDR opted to utilize the WVS App and Technology-aided micro-planning method for the remainder of the annual dog vaccination campaign in other departments of Haiti. As of February 2019, 330,000 dogs in Haiti were vaccinated using this method with periodic post-vaccination surveys finding coverage levels similar to what was reported here (31). For the first time in Haiti, data on vaccination counts combined with dog population information from targeted evaluations provides a clearer picture of what is required to achieve sustainable coverage levels. The use of smartphones allows every community in the country to be divided into strategic units for vaccination that can be followed longitudinally to ensure continued, homogenous coverage. In Haiti, the Traditional vaccination method was prone to missing large numbers of free-roaming dogs, and have so-far been unable to eliminate dog-mediated human rabies deaths. Reaching adequate dog vaccination coverages comes at a higher cost and more consideration for the logistical allocation of resources. However, if the goal of the program is to reach at least 70% of the susceptible dog population, then the Technology-aided vaccination method is a more cost-effective approach to vaccination. A redesign of how dog vaccination campaigns are implemented will assist in addressing coverage gaps and put Haiti on a path to rabies elimination.

## Data Availability Statement

The raw data supporting the conclusions of this article will be made available by the authors, without undue reservation.

## Ethics Statement

This project was deemed non-research by the CDC NCEZID IACUC and IRB offices and approved by authorities at MARNDR. All animals were handled by trained MARNDR staff. Informed consent was obtained from all participants prior to beginning household surveys.

## Author Contributions

This study design and protocol were developed by BM, AG, FLo, JB, EP, MN, MM, and RW. This study was performed by BM, AG, FLu, PD, KC, ME, MM, and RW. Data analysis was performed by BM, FLu, JC, and RW. This manuscript was written by BM, AG, MN, BM-F, and RW. Review and editing involved by FLo, JB, MN, BM-F, and MM. All authors contributed to the article and approved the submitted version.

## Funding

Merck Animal Health provided funding for distributing mobile phone SMS messages announcing the campaign.

## Author Disclaimer

This manuscript was prepared by the authors in their personal capacity. The findings and conclusions in this report are those of the authors and do not necessarily represent the official position of the Centers for Disease Control and Prevention, the Department of Health and Human Services, or the United States Government.

## Conflict of Interest

AG was project lead for the development of the WVS App as a part of employment for Mission Rabies. The remaining authors declare that the research was conducted in the absence of any commercial or financial relationships that could be construed as a potential conflict of interest.

## Publisher's Note

All claims expressed in this article are solely those of the authors and do not necessarily represent those of their affiliated organizations, or those of the publisher, the editors and the reviewers. Any product that may be evaluated in this article, or claim that may be made by its manufacturer, is not guaranteed or endorsed by the publisher.

## Abbreviations

CDC: US Centers for Disease Control and Prevention
MARNDR: Haiti Ministry of Agriculture and Rural Development (French?)
MSPP: Haiti Ministry of Public Health and Population (French?)
IBCM: integrated bite case management
IHSI: Institut Haitien de Statistique et d'Informatique
EA: enumeration area
H:D: human-to-dog-ratio
TA-after-traditional: technology-aided after traditional method
MVP: mobile vaccination point
DD: door-to-door vaccination
CVR, capture, vaccinate: and release vaccination
SRS: sight-resight.
